# Towards test guidelines and standards for advanced materials: scientific insights and recommendations from the MACRAMÉ project

**DOI:** 10.3389/ftox.2026.1895021

**Published:** 2026-09-01

**Authors:** Anna Pohl, Daniel Haase, Eric A. J. Bleeker, Samia Ouhajji, Klaus-Michael Weltring, Martin Wiemann, Alberto Katsumiti, Christian Seitz, Govind Gupta, Luis Mauricio Ortiz-Galvez, Tina Buerki-Thurnherr, Peter Wick, Thomas Eckart Exner, Blanca Suarez-Merino, Rayén Mentler, Tommaso Serchi, Katie Reilly, Iseult Lynch, Steffi Friedrichs, Elisabeth Heunisch

**Affiliations:** 1 AcumenIST SRL, Etterbeek, Belgium; 2 Federal Institute for Occupational Safety and Health (BAuA), Berlin, Germany; 3 Gesellschaft für Bioanalytik Münster e. V., Münster, Germany; 4 National Institute for Public Health and the Environment (RIVM), Bilthoven, Netherlands; 5 IBE R&D Institute for Lung Health gGmbH, Münster, Germany; 6 GAIKER Technology Centre, Basque Research and Technology Alliance (BRTA), Zamudio, Spain; 7 Empa – Swiss Federal Laboratories for Materials Science and Technology, Technology and Society Laboratory, St. Gallen, Switzerland; 8 Drug Discovery and Development Division, Patanjali Research Foundation, Haridwar, Uttarakhand, India; 9 Seven Past Nine GmbH, Schopfheim, Germany; 10 TEMAS Solutions GmbH, Hausen, Switzerland; 11 Luxembourg Institute of Science and Technology (LIST), Belvaux, Luxembourg; 12 School of Geography, Earth and Environmental Sciences, University of Birmingham, Birmingham, United Kingdom

**Keywords:** advanced materials, life cycle, MACRAMÉ informed recommendations, MACRAMÉ project, OECD Test Guidelines, safety testing, standardisation

## Abstract

The Horizon Europe Project MACRAMÉ developed twelve recommendations towards Test Guidelines and Standards for safety assessment of advanced materials (AdMas) that capture the identified needs in characterisation and identification, safety testing, transformation and release as well as information flow along the AdMas lifecycle. MACRAMÉ highlights challenges posed by complex structures and transformations of AdMas and AdMas-enabled products for regulation and industry drawn on research on graphene-related materials, carbon nanotubes and poly(lactic-co-glycolic) acid in realistic use cases. MACRAMÉ translates advances in characterisation, transformation and release assessment, and New Approach Methodologies into actionable recommendations and calls on regulators, funding bodies, researchers and industry to develop and validate fit-for-purpose test methods, standards and guidance with the aim to enable safe and sustainable deployment of AdMas for societal challenges.

## 1 Advanced materials challenge safety testing and risk assessment

Advanced Materials (AdMas) represent a rapidly evolving field, including materials with unique and novel structural features and functions. AdMas are used in a range of industrial sectors, including energy, electronics, water treatment, medical/health, and automotive applications (IAM4EU, 2024; European Research Council Executive Agency, 2026). The use of AdMas is even likely to rapidly increase based on the activities from the European Commission that is promoting the use of AdMas and providing research and innovation priorities in strategic areas (EU Commission, 2024). However, existing OECD Test Guidelines and standardised methods for safety assessment have been developed primarily for conventional chemicals and are slowly being adapted for nanomaterials. They often lack clear applicability to non-spherical particles, multi-component systems, or materials with dynamic behaviours, such as release during use or transformation *in situ* in products or the environment. Therefore, these methods are not always sufficient to capture the specific properties, exposure pathways, and transformation behaviours of some AdMas and AdMa-containing products (*e.g.* ECOS-BUND, 2023; Cassee et al., 2024). As a result, regulators may face challenges in assessing the safety of AdMa-enabled products and in making informed decisions across their life cycles, while companies may struggle to meet regulatory requirements efficiently. This may affect their willingness to invest in AdMas developments for current and future societal challenges (*e.g.* the green transition, replacing critical raw materials, or the digital transition) thus hampering Europe’s industrial competitiveness.

## 2 Policy options and implications: science-based recommendations from MACRAMÉ

Ensuring the safety and sustainability of AdMas requires fit-for-purpose and harmonised test methods, life cycle-based assessment approaches, and robust data management. Challenges from AdMas include the multitude of shapes, compositions and behaviours of AdMas, as well as their transformation and potential release along the life cycle of AdMa-containing products. Addressing these challenges requires regulatory and methodological adaptations. First, it is crucial to define and categorise AdMas to identify specific regulatory needs and adapt existing frameworks where necessary. Facilitating life cycle thinking in material design is essential to drive continued innovation in safe and sustainable materials. This also requires developing sector-specific methodologies and tools for Safe and Sustainable by Design (SSbD), along with incentives to encourage their use. Additionally, combined imaging and analytical approaches should be developed to quantify transformed AdMas. Finally, necessary steps forward include adjustment of harmonised test methods to make them applicable for AdMas, including New Approach Methodologies (NAMs), and ensuring the availability of harmonised test methods for sustainability assessments.

Safety research is essential to advance testing of materials and integrate scientific knowledge into regulatory risk assessment. The EU-funded MACRAMÉ Project (Horizon Europe, GA 101092686) has developed advanced characterisation methodologies to assess and predict the risks of advanced materials. MACRAMÉ focused on the testing of carbon-based materials, because of the specific challenges these pose with regard to detection in complex product and environmental matrices. The chosen graphene-related materials, carbon nanotubes and poly (lactic-co-glycolic) acid (PLGA) represent different categories of AdMas (*i.e*. 2D-materials, fibres, biodegradable polymers). Furthermore, they are incorporated in market-relevant products including water filters, batteries, car seats, spraying applications, and inhalable delivery. With these different applications in various product families, the different categories of AdMas challenge assessments in different regulatory frameworks, considering the form of the AdMas and their potential for release along their life cycle (Friedrichs et al., 2025). Based on these experiences, the project developed twelve recommendations to address gaps in AdMas characterisation and identification, safety testing, transformation and release, and information flow. These recommendations are grounded in collaborative research, combined with targeted consultations with stakeholders, including regulators, scientists, industry representatives (large companies, SMEs, testing laboratories) and experts from standardisation bodies and the OECD.

**FIGURE 1:**
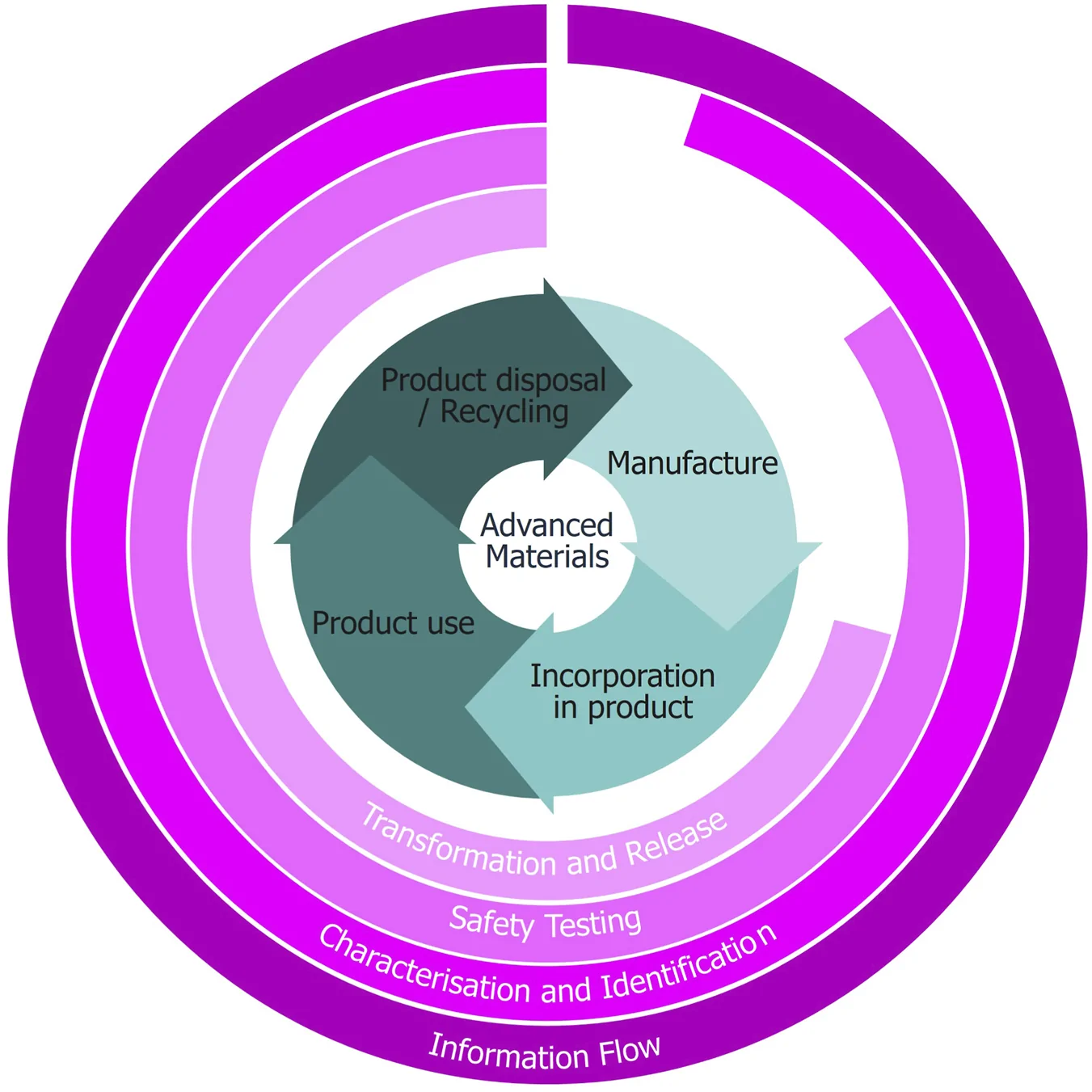
Overview of the overarching topics that are covered by the MACRAMÉ Informed Recommendations, highlighting the needs to be addressed towards Test Guidelines and Standards for AdMas along their life cycle (arrows in the centre).

The twelve recommendations, in brief, encompass the characterisation and identification of AdMas, in complex products and matrices including biological tissues, the safety testing of AdMas and AdMa-containing products, including the control of exposure dosage, their transformation and potential release along their life cycle as well as the transfer of information and data along the AdMas value chain (see Figure 1). Note that we consider life cycle stages of the entire life cycle of AdMa-containing products, which follow the typical stages of manufacturing, incorporation into a product, product use, and product disposal or recycling (as shown in Figure 1). We also consider transformation due to interactions with environmental compartments and organisms, during which a change of the physical and/or chemical properties of the AdMa can occur (Punz et al., 2025). The informed recommendations are listed in Table 1. For each recommendation, the expected output (*i.e.* guideline, guidance, standard, etc.) as well as the required actions are listed. In order to successfully implement the recommendations, collaborative actions from different key stakeholders and parties are needed (see Section 3). Elaborated descriptions of each individual recommendation (including the state of the art in the area) are provided as Supplementary Material.

**TABLE 1:** The MACRAMÉ informed recommendations.

The MACRAMÉ Informed Recommendations
Characterisation and Identification
1. Physico-Chemical Characterisation of Advanced Materials Output: Guidance to determine the minimal physico-chemical parameters for identification and characterisation of AdMa categories. Actions required: Proper categorisation of AdMas and the development of a decision tree to guide users in determining minimal characterisation requirements.
2. Detection and Quantification of Advanced Materials in Biological Matrices Output: Methods and guidance for the detection and quantification of AdMas and especially carbon-based materials in biological matrices. Actions required: Dedicated methods and standard operating procedures (SOPs) for existing microscopic and spectroscopic techniques and their combination in a coherent and versatile manner, supported by appropriate standards and reference controls.
3. Testing of Multi-Component Advanced Materials Output: Guidance on how to perform risk assessment for multi-component AdMas, including tailored approaches to identify, characterise and assess their potential synergistic or antagonistic effects. Actions required: Development of an integrated Approach to testing and Assessment (IATA) for multi-component AdMas, improved clarity in terminology and definitions, and a regulatory and scientific roadmap towards comprehensive, realistic and harmonised testing.
4. Predicting Toxic Potential of Fibres Based on Physico-Chemical Properties Output: Guidance for the determination of the toxic potential of fibres based on physico-chemical properties, *i.e.* critical dimensions, biopersistence and rigidity. Actions required: Development of test methods for critical dimensions of fibres and fibre bundles, rigidity and biopersistence via solubility/ dissolution rates. This needs to be supported by determining and establishing threshold values (including values for solubility/dissolution rates and rigidity linked to standardised measurement protocols).
Safety Testing
5. Sample Preparation for Safety Testing of Advanced Materials Output: Guidance on sample preparation for safety testing of AdMas specified for respective categories of AdMas, using specific test methods, biological systems, and/or endpoints. Actions required: Development of standard operating procedures (SOPs) for the adequate sample preparation in media needed for the different safety testing methods required for AdMas and their combination in a guidance.
6. Controlling and Describing the Dosimetry of Advanced Materials Output: Guidance on controlling and measurement of the dosimetry for adequate and accurate *in vitro* and *in vivo* testing for human and environmental toxicity of AdMas with realistic and relevant particle doses. Actions required: A quantitative description of delivered and cellular dose, as well as the development of quantitative high-resolution methods based on a combination of microscopic, analytical, and (mass) spectroscopic methods.
7. Testing and Assessment Strategies for Profound Risk Assessment of Advanced Materials Depending on Identified Exposure Points Output: An integrated Approach to testing and Assessment (IATA) for AdMas that guides risk assessors towards essential tests to be performed on an AdMa and/or AdMa-containing product, depending on and considering the relevant identified exposure points together with the AdMa’s and/or product’s associated fate and mobility. Actions required: Development of a set of reliable and robust standards and reference materials for toxicity testing.
Transformation and Release
8. Identify Critical Steps in the Life Cycle where (Transformed) Advanced Materials are Released Output: Guidance on an optimised approach to identify where (transformed) AdMas are released along their life cycle to inform a more dedicated risk assessment. Actions required: Development of a guidance on conducting material flow analysis (MFA) studies as a pre-experimental approach to identify form-specific releases from the specific product application, including criteria for selecting the approach (static versus dynamic) and for reporting and interpreting the outcomes.
9. Standardised Release Testing of Advanced Materials along their Life Cycle Output: Standardised release tests that adequately mimic relevant exposure scenarios along AdMa life cycles to enable qualitative and quantitative analysis of (potential) releases from AdMas and AdMas-containing products along their life cycle. This should include detection and characterisation methods for released (fragments of) materials. Actions required: Identification of specific intentional and unintentional transformation processes during the life cycle for which no (standardised) release test currently exists, and further validation and standardisation of existing release tests to ensure their applicability for AdMas.
10. Influence of Physical and Chemical Transformations in the Environment on Risk Profiles of Advanced Materials Output: Guidance on how to test the transformations of the physical form of AdMas occurring in the environment. Actions required: Performing a scoping review to identify the most important transformation processes in relevant environmental compartments, followed by the development of test methods, potentially including modelling tools to predict physical transformations.
Information Flow
11. Life Cycle Information as part of Advanced Material Characterisation Output: Guidance for a provenance-based documentation of the AdMa life cycle, including its environment and covering information on each individual life cycle stage and transformations between stages, improving interpretability and comparability of safety assessments. Actions required: A data documentation framework and corresponding standards that defines information requirements for the description of the life cycle history and fate of an AdMa complementing the identification and characterisation of the AdMa at a specific life cycle stage previous to safety testing.
12. Sharing of Safety-relevant Information on Advanced Materials along their Life Cycle Output: Improved digital management and sharing approaches with corresponding reporting and (meta)data standards for safety-relevant information on AdMas including transformations along the life cycle that ensure that the data are findable, accessible, interoperable, and reusable (FAIR). Actions required: The development of harmonised and standardised sharing tools and the creation of guidance on how to structure data collection and sharing along the AdMas’ value chain and life cycle.

## 3 Call for action

The MACRAMÉ project encourages the European Commission, OECD Member Countries and all involved stakeholders to collaboratively support the advancement of Test Guidelines and standards as identified in the MACRAMÉ Informed Recommendations by implementing the following actions:

- Funding agencies in Europe and other OECD Member Countries to fund targeted research and method development, optimisation and validation to accelerate the development of Test Guidelines and standards for AdMas.
- Regulatory agencies and standardisation bodies (OECD/ISO/CEN) to initiate the development of OECD Test Guidelines, ISO/CEN standards, and/or guidance documents in the twelve identified areas to ensure they are fit-for-purpose for AdMas and AdMa-containing products.
- Researchers to support the development of Test Guidelines and standards by translating their developed methodologies into respective Standard Operating Procedures or protocols that allow transfer to other laboratories.
- Industry (including contract research organisations, testing laboratories, and service providers) to aid the development and validation of test methods by providing information (*e.g.* on realistic use/end-of-life scenarios) and testing experiences.

## 4 Conclusion

The MACRAMÉ project highlights that AdMas require fit-for-purpose, harmonised and life cycle–oriented approaches for safety assessment. With the twelve Informed Recommendations the MACRAMÉ project aims to address the regulatory challenges in assessing AdMas safety and sustainability and facilitate companies to meet regulatory requirements efficiently. This will support European competitiveness in exploiting the full potential of the use of AdMas to address current and future societal challenges (*e.g.* the green transition, replacing critical raw materials, or the digital transition).
