# Supplementary material for: Towards test guidelines and standards for advanced materials: scientific insights and recommendations from the MACRAMÉ project
Source: Front Toxicol. 2026 Sep 1;8:1895021. doi: 10.3389/ftox.2026.1895021 (PMC13574966; doi:10.3389/ftox.2026.1895021)
Supplement: Supplementary file 1 [file DataSheet1.pdf]

Supplementary Material

MACRAMÉ Informed Recommendations elaborations.

1 Physico-Chemical Characterisation of Advanced Materials 2
2 Detection and Quantification of Advanced Materials in Biological Matrices 4
3 Testing of Multi-Component Advanced Materials 7
4 Predicting Toxic Potential of Fibres Based on Physico-Chemical Properties 10
5 Sample Preparation for Safety Testing of Advanced Materials 13
6 Controlling and Describing the Dosimetry of Advanced Materials 16
7 Testing and Assessment Strategies for Profound Risk Assessment of Advanced Materials Depending on Identified Exposure Points 18
8 Identify Critical Steps in the Life Cycle where (Transformed) Advanced Materials are Released 20
9 Standardised Release Testing of Advanced Materials along their Life Cycle 22
10 Influence of Physical and Chemical Transformations in the Environment on Risk Profiles of Advanced Materials 24
11 Life Cycle Information as Part of Advanced Material Characterisation 27
12 Sharing of Safety-relevant Information on Advanced Materials along the Life Cycle 30

1 Physico-Chemical Characterisation of Advanced Materials

Recommendation

Develop guidance to determine the minimal physico-chemical parameters for identification and characterisation of advanced materials categories.

Issue

Physico-chemical characterization provides detailed information regarding the physical properties, composition and microscopic structure of a material. This material characterization helps to identify the intrinsic properties of a material, such as shape, size, flammability, and reactivity. Understanding these properties is critical for recognizing potential hazards associated with the material and performing accurate risk assessments, as experiences with nanomaterials have shown.

State-of-the-art and resources to be considered

A category of Advanced Materials (AdMas) for which an identification of essential physico-chemical characterisation parameters was performed is the graphene family of two-dimensional materials (GR2M). For GR2M three essential physico-chemical properties were identified, *i.e.* number of layers, carbon/oxygen ratio and average lateral dimensions (Wick et al. 2014). The Advanced Carbon Council, formerly known as Graphene Council expanded this set to characterize GR2M regardless of production method and raw materials used in its Graphene Classification Framework (GCF). The GCF lists the 19 key graphene material characteristics that should be reported in the technical data sheet for commercial application. Of these 19, nine are considered the minimum set of details necessary to provide a one-sentence shorthand description (syntax) for any type or form of GR2M. These are layer count, lateral size, particle shape, oxidation, functionalisation, form, solvent (if dispersed), production method, and raw material. The GCF has now been finalised and published as an ISO Technical Specification in July 2025 (ISO/TS 9651). Next to the minimum set of relevant characteristics and the syntax, this standard comprises methods of testing for each characteristic, a range of the expected measurement values for benchmarking, and to define a standardized technical data sheet. These methods are at least in part applicable to even more complex AdMas, which contain GR2M embedded in a variety of organic matrices as investigated in MACRAMÉ. A report by ECHA also stresses the need for thorough characterization of each type of graphene and 2D material including their production and fate, but proposes minimal requirements with chemical composition, structure, lateral size, and number of layers as the only mandatory characteristics. However, the ECHA report also mentions other important characteristics. This merely demonstrates the current lack of agreement in the scientific and regulatory communities even for this well-investigated AdMa class (European Chemicals Agency 2022). For other and especially emerging AdMas, such lists of important characteristics do not exist at all.

For other AdMas, no GCF-like evaluation scheme has been conducted, although the European REACH Regulation aims to characterise nanoforms of substances (potentially including certain AdMas) by a minimum of particle size distribution, shape, volume specific surface area and surface chemistry (EU Commission 2018). Nevertheless, given the broad scope of AdMas as described by the OECD (OECD 2023a), defining a universal set of requirements for all categories of AdMas is likely improbable. The lack of (certified) reference materials for AdMas further complicates the issue. It might, therefore, be more practical to establish requirements tailored to specific categories of materials. Yet, as shown by the large variety within the realm of 2D materials, defining specific categories remains very

challenging. The ongoing ECHA-funded project "Nanomaterial Risk Assessment: A Regulatory Way Forward for Sameness and Grouping Approaches" may provide valuable insights into the grouping of AdMas. Similar challenges also exist for multi-component materials (see Recommendation 3).

In-depth characterization of AdMas could focus on critical properties such as size, shape, chemistry, physical attributes, stability, dissolution rate and oxidation state. Using an appropriate testing medium during dispersion and characterization is key to obtaining accurate and meaningful results not only for characterization but also for the application of toxicological *in vitro* methods. The OECD TG 124 (OECD 2022) and OECD TG 125 (OECD 2023b), for example, provide comprehensive methods for determining the volume-specific surface area of manufactured nanomaterials and the nanoparticle size and size distribution (1-1000 nm), respectively. However, although these TGs may be applicable, they have not been validated for use with AdMas, so additional work in this area may be required. In addition, ISO Technical Committee (TC) 229 is actively developing international standards for nanotechnologies, which include some guidance on the physical-chemical characterization of AdMas. It is recommended to develop practical guidance that is accessible to Small & Medium Enterprises (SMEs). Participation of SMEs in the development of these standards will ensure their relevance and effectiveness.

Output / product(s) envisioned and action(s) required

The envisioned product is a guidance to identify the minimal morphological information needed for physico-chemical characterisation of AdMas. To effectively achieve this, it is imperative to properly categorise AdMas. In addition, a decision tree to guide users appears needed to determine the minimal characterization requirements (based on material type, use, purpose and exposure), taking the purpose of the assessment into account.

References

EU Commission (2018): Commission Regulation (EU) 2018/1881 of 3 December 2018 amending Regulation (EC) No 1907/2006 of the European Parliament and of the Council on the Registration, Evaluation, Authorisation and Restriction of Chemicals (REACH) as regards Annexes I, III,VI, VII, VIII, IX, X, XI, and XII to address nanoforms of substances, http://data.europa.eu/eli/reg/2018/1881/oj. Available online at http://data.europa.eu/eli/reg/2018/1881/oj.

European Chemicals Agency (2022): Assessment of the potential impact of graphene, graphene oxide and other 2D materials on health, and the environment. European Chemicals Agency. Available online at https://data.europa.eu/doi/10.2823/551125.

ISO/TS 9651, 2025: Nanotechnologies — Classification framework for graphene-related 2D materials. Available online at https://www.iso.org/standard/84232.html.

OECD (2022): Test No. 124: Determination of the Volume Specific Surface Area of Manufactured Nanomaterials. Edited by OECD Guidelines for the Testing of Chemicals, Section 1. OECD Publishing. Paris. Available online at https://doi.org/10.1787/abb72f8f-en.

OECD (2023a): Advanced Materials: Working Description. Edited by OECD Series on the Safety of Manufactured Nanomaterials and other Advanced Materials. OECD Publishing. Paris. Available online at https://doi.org/10.1787/4b5ba38d-en.

OECD (2023b): Test No. 125: Nanomaterial Particle Size and Size Distribution of Nanomaterials. Edited by OECD Guidelines for the Testing of Chemicals, Section 1. OECD Publishing. Paris. Available online at https://doi.org/10.1787/af5f9bda-en.

Wick, Peter; Louw-Gaume, Anna E.; Kucki, Melanie; Krug, Harald F.; Kostarelos, Kostas; Fadeel, Bengt et al. (2014): Classification framework for graphene-based materials. In *Angewandte Chemie (International ed. in English)* 53 (30), pp. 7714–7718. DOI: 10.1002/anie.201403335.

2 Detection and Quantification of Advanced Materials in Biological Matrices

Recommendation

Develop methods and guidance for the detection and quantification of AdMas, especially carbon-based materials in biological matrices.

Issue

The lack of regulatory guidance on the detection and quantification of AdMas upon contact with biological matrices causes great uncertainty. This makes it impossible to fully apply regulation and guidelines, which would require detection and/or quantification of materials for classification purposes. This is further exacerbated for carbon-based materials, for which identification is at least severely hindered, especially if a material with carbonaceous composition is to be detected within biological matrices.

State-of-the-art and resources to be considered

Many of the qualitative and quantitative techniques that are used for the detection and quantification of materials and AdMas are based on the different physico-chemical properties of the materials, as compared to the biological background. For example, Secondary Ion Mass Spectrometry (SIMS) is a powerful technique that allows for the analysis of surfaces and therefore may be applied to solid samples. SIMS enables the identification of elemental composition, isotopic ratios, and molecular structures with high spatial precision. There are also some SIMS techniques that make use of lower energy and allow the detection of small molecules. These can be useful as some AdMas are made of polymers, which could be detected using *e.g.*, Time-of-Flight Secondary Ion Mass Spectrometry (ToF-SIMS). However, SIMS requires for the sample to contain some element that can be distinguished from the biological background. Thus, the detection of AdMas composed only of elements common in biological samples (*e.g.*, C, N, O) is difficult with this technique. Similar issues hamper the use of other methods. In the past, nano-SIMS was successfully used for the detection of carbon nanotubes (CNTs) embedded within a biological matrix, but only by using traces of non-carbon catalyser elements used in the production of the CNTs.

When it comes to detection and quantification of AdMas in biological fluids and in cell culture media, there are other types of limitations that need to be considered. In principle, liquid samples are subjected to the same limitations described above for solid samples. However, strategies aiming at isolation and separation of the dispersed materials can be deployed. In this case, it is important that the isolation method achieves separation of the AdMas in a quantitative and reproducible way. It is also important that proper blanks and controls are included within the experimental protocol. Dynamic Light Scattering (DLS) and Nanoparticle Tracking Analysis (NTA) are among the most available and used techniques for the detection of particle size distribution and quantification of nanomaterials and AdMas in aqueous media. They are relatively accessible and simple to use and provide data on the hydrodynamic diameter of particles. Therefore, they suffer some severe limitations because they assume that the shape of the AdMas is spherical, thus results with AdMas that are non-spherical are not reliable.

There is also significant interference from other components present in the cell culture media. These methods work best with ultrapure water, but this is not representative of the particle behaviour in cell

culture media. Furthermore, the dispersion to be analysed with DLS or NTA needs to be "sufficiently stable", which is an arbitrary definition. Dispersion stability depends, along with other parameters, on the nature of the AdMa, on temperature, pH, ionic strength, concentration and presence of biomolecules (*e.g.*, serum, lipids). These parameters will dictate the behaviour of the material in dispersion and determine aggregation and sedimentation.

To detect, visualize and identify particles in cells and tissue-like cell cultures, numerous microscopy-based and spectroscopy-based methods have been developed during the last decade. Darkfield Microscopy (DFM), Hyperspectral Imaging (HSI), Confocal Raman Microscopy (CRM), Laser Scanning Microscopy (LSM), Infrared Microscopy (IRM) or ToF-SIMS are all useful methods, which not only detect but, within limits, can also identify materials in cells and tissues (Veith et al. 2018; Vennemann et al. 2017; Seiffert et al. 2022). This is increasingly relevant for AdMas containing mixed and/or carbonaceous materials, *e.g.*, GR2M or CNTs (Chaloupková et al. 2023). Here, CRM or IRM may help identify the materials in question and could even quantify the number of inclusions, serving as a semi-quantitative approach. Analytical methods such as laser ablation inductively coupled plasma mass spectroscopy (LA-ICP-MS) can quantify at least metal-based AdMas in single cells. Nevertheless, the quantitative analysis of AdMas in cells and tissues is still an issue and further development is needed to cover the whole spectrum of materials.

The proposed list is not exhaustive, as many other alternative techniques could be applied. However, many of these methods are not yet routinely available in Contract Research Organisations (CROs), particularly those conducting ecotoxicity studies.

Output / product(s) envisioned and action(s) required

Several advanced microscopic and spectroscopic techniques are already available or under development that enable the detection and quantification of AdMas, particularly carbon-based materials, within biological matrices (a non-exhaustive set was presented above). However, these methods often rely on expensive and specialised equipment and require trained personnel and dedicated procedures that are not routinely available in most laboratories or CROs, especially those conducting ecotoxicity studies.

There is therefore an urgent need to develop dedicated methods and Standard Operating Procedures (SOPs), supported by appropriate standards and reference controls. Existing microscopic techniques that allow the detection and quantification of AdMas within biological matrices should be combined in a coherent and versatile manner, and their use should be described in a guidance document. This is particularly important for carbon-based materials, which remain difficult to detect and quantify with currently available methods.

Wherever applicable, high-resolution techniques such as confocal Raman spectroscopy, transmission electron microscopy (TEM), and hyperspectral imaging may be employed for carbon-based AdMas. However, an adequate test guideline still needs to be developed, linking these methods to their respective SOPs. Methods for carbon-based AdMas should also be validated for their detection capability using ^{13}C-labelled materials (stable isotope tracers). This recommendation is closely linked to Recommendation 2, which addresses the detection and quantification of AdMas and their transformed forms in released fragments.

References

Chaloupková, Zuzana; Žárská, Ludmila; Belza, Jan; Poláková, Kateřina (2023): Label-free detection and mapping of graphene oxide in single HeLa cells based on MCR-Raman spectroscopy. In *Analytical methods : advancing methods and applications* 15 (42), pp. 5582–5588. DOI: 10.1039/d3ay01122d.

Seiffert, Svenja B.; Vennemann, Antje; Nordhorn, Ilona D.; Kröger, Sabrina; Wiemann, Martin; Karst, Uwe (2022): LA-ICP-MS and Immunohistochemical Staining with Lanthanide-Labeled Antibodies to Study the Uptake of CeO2 Nanoparticles by Macrophages in Tissue Sections. In *Chemical research in toxicology* 35 (6), pp. 981–991. DOI: 10.1021/acs.chemrestox.1c00433.

Veith, Lothar; Böttner, Julia; Vennemann, Antje; Breitenstein, Daniel; Engelhard, Carsten; Meijer, Jan et al. (2018): Detection of ZrO_2 Nanoparticles in Lung Tissue Sections by Time-of-Flight Secondary Ion Mass Spectrometry and Ion Beam Microscopy. In *Nanomaterials (Basel, Switzerland)* 8 (1). DOI: 10.3390/nano8010044.

Vennemann, Antje; Alessandrini, Francesca; Wiemann, Martin (2017): Differential Effects of Surface-Functionalized Zirconium Oxide Nanoparticles on Alveolar Macrophages, Rat Lung, and a Mouse Allergy Model. In *Nanomaterials (Basel, Switzerland)* 7 (9). DOI: 10.3390/nano7090280.

3 Testing of Multi-Component Advanced Materials

Recommendation

Develop and establish guidance on how to perform risk assessment for multi-component AdMas, including tailored approaches to identify, characterise and assess their potential synergistic or antagonistic effects.

Issue

Multi-component AdMas may pose unintended risks arising from the interactions between their individual constituents. Such synergistic or antagonistic effects challenge the current regulatory preparedness, since existing frameworks (particularly REACH) typically address individual components or classify multi-component materials as mixtures without requiring integrated hazard testing. This results in uncertainties for manufacturers regarding testing strategies, classification, and registration.

Moreover, definitions and terminology for multi-component nanomaterials (MCNMs) or AdMas (*e.g.* nanocomposites, hybrid materials, nanocarriers) remain inconsistent across regulatory and scientific contexts, complicating comparability and risk communication.

State-of-the-art and resources to be considered

Recent analyses (*e.g.* (Hunt et al. 2025)) have highlighted that current REACH provisions may not fully capture the complexity of MCNMs. Although REACH includes both mono- and multi-constituent substances, its testing requirements do not explicitly account for potential mixture or interfacial effects arising within an integrated material system.

For hazard and risk assessment, testing of individual components alone may underestimate the actual hazard profile of the combined system. A standardised *mixture assessment factor* can compensate for some uncertainty, yet dedicated testing strategies addressing interactions among components are needed. OECD GD 23 (OECD 2019) provides a general framework for assessing chemical mixtures, but it does not capture the physico-chemical and dynamic interactions specific to multi-component AdMas.

A recent review of research gaps regarding MCNMs for hazard assessment identified significant knowledge gaps related to their environmental fate, transformations upon exposure to new environments, and potential adverse effects on organisms and ecosystems. More detailed assessments of release, transformation, and bioavailability of the different components (particularly in biologically relevant media) may provide further insight into mixture toxicity and interaction potential.

Stakeholder feedback emphasised several critical elements for improving the robustness and applicability of the recommendation:

- Structural configuration should be considered explicitly. In core–shell or layered architectures, the shell or surface coating may largely determine hazard and exposure potential.
- Dissolution and leaching studies in biologically relevant media (*e.g.* pulmonary or gastrointestinal fluids) are required to identify released species and explore their potential (eco)toxicity.

- Further testing of dissolution and leaching in lysosomal fluid (the so-called "cellular dustbin") should be included to better understand degradation and transformation processes under realistic biological conditions. This is important not only for multi-component materials.
- Transformation and bioavailability of each component must be assessed under realistic environmental and biological conditions, including coating loss, aggregation, or re-precipitation effects.
- Integration with Integrated Approaches to Testing and Assessment (IATAs) should be promoted to support mixture toxicity testing and exposure-driven risk evaluation.
- Testing should aim to enable understanding of whether the components of multi-component AdMas interact additively or synergistically, and whether specific structural arrangements (*e.g.*, core–shell versus chemical doping) influence the potential for such interactions.

An emerging set of SOPs for dissolution testing of MCNMs (Di Cristo et al. 2024) offers an important first step toward harmonised assessment. Building on these efforts, further testing of the leachates and transformed products will be essential to elucidate potential synergistic or antagonistic effects among components.

At the regulatory level, alternative approaches to registration could be explored. Instead of separate registration of each constituent, AdMas with strong structural or functional integration (*e.g.*, functionalised nanomaterials) could be registered as *single entities with multiple constituents*. This approach would align with current practices for functionalised nanomaterials and better reflect their integrated hazard and exposure characteristics.

At the regulatory level, clarity of terminology and framework design is essential to establish consistent requirements for multi-component AdMas. Suggestions on how to address MCNMs and AdMas include following the approach used for functionalised nanomaterials, in which the major component is registered as the primary entity while additional components contributing to functionalisation are considered integral parts of the material, even if they consist of several substances or elements.

Relevant initiatives and resources to consider, include:

- OECD GD 23 (OECD 2019) on chemical mixtures;
- Outcomes of the Horizon Europe project HARMLESS, which provides concepts for integrated hazard and risk assessment of complex materials;
- Activities conducted under MACRAMÉ and related Horizon Europe projects (*e.g.* SUNSHINE) for the development of IATAs and SOPs supporting multi-component materials.

Output / product(s) envisioned and action(s) required

The envisioned outcome is a guidance document defining how to design, perform and interpret testing of multi-component AdMas, incorporating interaction mechanisms, leachate testing, and transformation processes for both AdMas and AdMa-containing products.

To achieve this, the following actions are required:

- Develop an IATA for multi-component AdMas that combines physico-chemical characterisation, dissolution, transformation, and hazard endpoints.

- Provide terminological and definitional clarity to improve regulatory predictability under REACH (EU Commission 2006), CLP (EU Commission 2008) and related frameworks.
- Promote harmonisation and standardisation efforts under OECD and ISO to align methodologies for complex materials.
- Establish links with existing and emerging projects (*e.g.* HARMLESS, MACRAMÉ, SUNSHINE, CHIASMA) to ensure methodological coherence and avoid duplication.

Ultimately, such guidance should deliver a regulatory and scientific roadmap that enables comprehensive, realistic, and harmonised testing of multi-component AdMas. This should then ensure comparability, cost-efficiency, and fostering Safe-and-Sustainable-by-Design innovation.

References

Di Cristo, Luisana; Keller, Johannes G.; Leoncino, Luca; Marassi, Valentina; Loosli, Frederic; Seleci, Didem Ag et al. (2024): Critical aspects in dissolution testing of nanomaterials in the oro-gastrointestinal tract: the relevance of juice composition for hazard identification and grouping. In *Nanoscale advances* 6 (3), pp. 798–815. DOI: 10.1039/d3na00588g.

EU Commission (2006): Commission Regulation (EC) No 1907/2006. of the European Parliament and of the Council of 18 December 2006 concerning the Registration, Evaluation, Authorisation and Restriction of Chemicals (REACH), establishing a European Chemicals Agency, amending Directive 1999/45/EC and repealing Council Regulation (EEC) No 793/93 and Commission Regulation (EC) No 1488/94 as well as Council Directive 76/769/EEC and Commission Directives 91/155/EEC, 93/67/EEC, 93/105/EC and 2000/21/EC. Available online at http://data.europa.eu/eli/reg/2006/1907/oj.

EU Commission (2008): Regulation (EC) No 1272/2008. of the European Parliament and of the Council of 16 December 2008 on classification, labelling and packaging of substances and mixtures, amending and repealing Directives 67/548/EEC and 1999/45/EC, and amending Regulation (EC) No 1907/2006 (Text with EEA relevance). Available online at http://data.europa.eu/eli/reg/2008/1272/oj.

Hunt, Neil; Kestens, Vikram; Rasmussen, Kirsten; Badetti, Elena; Soeteman-Hernández, Lya G.; Oomen, Agnes G. et al. (2025): Regulatory preparedness for multicomponent nanomaterials: Current state, gaps and challenges of REACH. In *NanoImpact* 37, p. 100538. DOI: 10.1016/j.impact.2024.100538.

OECD (2019): Guidance Document 23 on Aquatic Toxicity Testing of Difficult Substances and Mixtures. Edited by OECD Series on Testing and Assessment. OECD Publishing.

4 Predicting Toxic Potential of Fibres Based on Physico-Chemical Properties

Recommendation

Develop guidance for the determination of the toxic potential of fibres based on physico-chemical properties *i.e.*, critical dimensions, biopersistence and rigidity.

Issue

It is well known that inhalation of naturally occurring mineral fibres such as asbestos can lead to the development of lung diseases and even incurable mesothelioma. The same is true for manufactured (advanced) fibres if they have specific properties, *i.e.*, specific dimensions, biopersistence and rigidity. Establishing relevant threshold values for these properties is pivotal for Safe-and-Sustainable-by-Design (SSbD) as well as for regulatory testing of fibre-like AdMa.

State-of-the-art and resources to be considered

Fibres have a fibre-specific toxicity if they have specific dimensions, are biopersistent and are rigid (Rittinghausen et al. 2014). Determining these properties can help determine if a specific fibre material can lead to lung diseases or mesothelioma, without requiring extensive (toxicity) testing. The critical dimensions according to the World Health Organisation (WHO) counting rules are set to a width of less than 3 µm, a length greater than 5 µm, and a minimum aspect ratio of 3:1. The critical dimensions can be either fulfilled by single fibres or fibre-shaped bundles (agglomerates, aggregates). Standardised and harmonised methods for the size determination of fibres are available (*e.g.*, OECD TG 125 (OECD 2023)).

The biopersistence is assessed as a combination of non-clearance (linked to the length and rigidity of the fibres) and the biodurability defined by a low solubility/dissolution rate in relevant biological fluids. Standardised and harmonised methods for the determination of solubility and dissolution rate are available or are under development (OECD TG 105 (OECD 1995), OECD TGP project 1.5). The rigidity is linked to the failure of the macrophages to bend and therefore incorporate the fibre leading to frustrated phagocytosis (Broßell et al.). The diameter of a fibre (bundle) may provide a proxy for rigidity, but harmonised/standardised methods are lacking for this property. Alternatively, mapping of defects may be predictive of AdMa toxicity, and may provide insights into the rigidity as defects represent breakage points and correlate with mechanical strength (Sammalkorpi et al. 2004).

In addition to these three parameters, surface-related properties such as surface chemistry, charge, functionalisation, and reactivity can also significantly influence fibre toxicity. These properties affect how fibres interact with biological systems, including adsorption of proteins and biomolecules, induction of oxidative stress, and membrane interactions. Therefore, it is recommended to explicitly include surface properties within the scope of characterisation to better predict toxic potential and ensure comprehensive assessment of fibre hazards.

For fibrous nanomaterials, such as carbon nanotubes, the proposed Occupational Exposure Limit (OEL) value is 0.01 fibres/cm^3 (Mihalache et al. 2017). Detecting nanofibres at the concentration level of 0.01 fibres/cm^3 would require evaluation of >10^5 micrograph images and is difficult to conduct (Koivisto et al. 2018) and therefore requires automated image acquisition and automated image analysis supported by AI tools (Peters et al. 2026).

Output / product(s) envisioned and action(s) required

To support material developers in testing if their fibres are likely to be toxic, a guidance document leading users stepwise through the determination of the critical dimensions, biopersistence and rigidity is needed. To support this development the following activities are required.

- **Critical dimensions**: the critical values for the fibre width and length are well established and standardised measurement procedures are established (*e.g.*, OECD TG 125 (OECD 2023)). A challenge is the measurement of fibre bundles. Development of sample preparation and measurement procedures is needed.
- **Biopersistence**: an *in vivo* half-life threshold that induces no fibrosis or tumours needs to be determined and correlated to solubility/dissolution rates (Madl and O'Neill 2022). Based on this threshold value, related values for solubility/dissolution rates need to be established and linked to standardised measurement protocols, that also specify the relevant test parameters (fluids, pH, temperature, timepoints, etc.).
- **Rigidity**: a threshold value for critical rigidity, inducing frustrated phagocytosis, needs to be determined and established. For the determination of the threshold value, the macrophage assay is a valuable instrument (Broßell et al. 2026) that needs to be further developed and standardised. Additional methods for the determination of rigidity need to be developed; these can be *in vitro* methods (*e.g.*, macrophage assay) as well as physico-chemical testing approaches (*e.g.*, using electromechanical resonance, micromanipulation, or defect determination via Raman and Transmission Electron Microscopy (TEM), providing direct visual evidence of defects).

References

Broßell, D.; Meyer-Plath, A.; Kämpf, K.; Plitzko, S.; Wohlleben, W.; Stahlmecke, B. et al.: A human risk banding scheme for high aspect-ratio materials: Chapter 7. In : in: Synthetic Nano- and Microfibers / R. M. Wagterveld, J. C.M. Marijnissen, L. Gradon, A. Moskal (Eds.) Leeuwarden: Wetsus, 2020. pages: 55-79, Project number: F 2480, pages: 55-79.

Broßell, Dirk; Meyer-Plath, Asmus; Gräb, Oliver; Heunisch, Elisabeth; Kämpf, Kerstin; Haase, Andrea; Wiemann, Martin (2026): Macrophages bend long fibres with flexural rigidity lower than 3 mN·nm2 to avoid frustrated phagocytosis. In *Particle and fibre toxicology* 23 (1). DOI: 10.1186/s12989-026-00666-9.

Koivisto, Antti Joonas; Bluhme, Anders Brostrøm; Kling, Kirsten Inga; Fonseca, Ana Sofia; Redant, Emile; Andrade, Flavia et al. (2018): Occupational exposure during handling and loading of halloysite nanotubes – A case study of counting nanofibers. In *NanoImpact* 10, pp. 153–160. DOI: 10.1016/j.impact.2018.04.003.

Madl, Amy K.; O'Neill, Heidi C. (2022): Fiber biodurability and biopersistence: historical toxicological perspective of synthetic vitreous fibers (SVFs), the long fiber paradigm, and implications for advanced materials. In *Critical reviews in toxicology* 52 (10), pp. 811–866. DOI: 10.1080/10408444.2022.2154636.

Mihalache, Raluca; Verbeek, Jos; Graczyk, Halshka; Murashov, Vladimir; van Broekhuizen, Pieter (2017): Occupational exposure limits for manufactured nanomaterials, a systematic review. In *Nanotoxicology* 11 (1), pp. 7–19. DOI: 10.1080/17435390.2016.1262920.

OECD (1995): Test No. 105: Water Solubility. With assistance of OECD Guidelines for the Testing of Chemicals, Section 1. Edited by OECD Publishing. Available online at https://doi.org/10.1787/9789264069589-en.

OECD (2023): Test No. 125: Nanomaterial Particle Size and Size Distribution of Nanomaterials. Edited by OECD Guidelines for the Testing of Chemicals, Section 1. OECD Publishing. Paris. Available online at https://doi.org/10.1787/af5f9bda-en.

Peters, Torben; Schumann, John; Kämpf, Kerstin; Meyer-Plath, Asmus (2026): AI-assisted recognition of fibre instances in scanning electron microscope images for aerosol characterization and exposure assessment. In *Powder Technology* 469, p. 121909. DOI: 10.1016/j.powtec.2025.121909.

Rittinghausen, Susanne; Hackbarth, Anja; Creutzenberg, Otto; Ernst, Heinrich; Heinrich, Uwe; Leonhardt, Albrecht; Schaudien, Dirk (2014): The carcinogenic effect of various multi-walled carbon nanotubes (MWCNTs) after intraperitoneal injection in rats. In *Particle and fibre toxicology* 11, p. 59. DOI: 10.1186/s12989-014-0059-z.

Sammalkorpi, M.; Krasheninnikov, A.; Kuronen, A.; Nordlund, K.; Kaski, K. (2004): Mechanical properties of carbon nanotubes with vacancies and related defects. In *Phys. Rev. B* 70 (24). DOI: 10.1103/PhysRevB.70.245416.

5 Sample Preparation for Safety Testing of Advanced Materials

Recommendation

Develop guidance on sample preparation for safety testing of AdMas specified for respective categories of AdMas, using specific test methods, biological systems, and/or endpoints.

Issue

AdMas generally need to be tested in their particulate form to avoid loss of their advanced properties. This raises challenges for sample preparation, a critical step in analytics and toxicity testing. Generally, the aim is to test the 'as produced' material[1], while the material needs some preparation steps to allow characterisation of the material itself (*e.g.*, by electron microscopy), or a proper dosing in the test system (*e.g.*, by using an aqueous stock dispersion), potentially changing the properties of the material. To further complicate the issue, sample preparation may differ depending on the tested endpoints. Physico-chemical characterisation of the 'as produced' material, for example, may need different sample preparation (*e.g.*, for analysis under vacuum such as in transmission electron microscopy analysis) from that for toxicity testing which may require dosing as an aerosol or as a liquid dispersion. (*e.g.*, dry versus wet samples).

State-of-the-art and resources to be considered

While some AdMas may have their own specific (additional) challenges and, issues,potential solutions for nanomaterials have been identified and published by the OECD (OECD 2012). A considerably adapted update of this document has been published recently (OECD 2025). While some of the potential solutions identified may be applicable to AdMas as well, the updated document remains focused on nanomaterials. As a rule of thumb sample preparation should be kept to a minimum, *e.g.*, when material is purchased as a dry powder it is usually recommended to keep it as a dry powder, unless a specific test system or characterisation method requires an aqueous dispersion (see also (OECD 2025)).

The often-complex multi-component nature of AdMas poses challenges for their physico-chemical characterisation. Additional difficulties arise when the materials need to be characterised within complex matrices (*e.g.*, soil, tissues, cells), especially when background chemistry is high (*e.g.*, carbon-based materials in carbon-rich tissues, see also Recommendation 2). Measurement techniques for physico-chemical parameters may require steps that may already change (some of) the physico-chemical parameters of the material (*e.g.*, certain electron microscopy techniques require the material to be in a vacuum).

For *in vitro* toxicity testing with cell models most challenges are related to the fact that many tests require exposure in an aqueous medium. This generally requires methods to disperse the (particulate) materials. Depending on the test system and particle size, dispersion stability can be the result of an interaction between the dispersed substance and the specific (aqueous) medium used, potentially leading to alterations of the tested material and/or its toxicological profile. Methods and equipment for the exposure of the biological system to dry materials have been developed. This appears particularly

---

[1] Note that 'as produced' may also encompass 'as released' at different steps in the life cycle of (products containing) AdMas.

relevant in the case of Air-Liquid-Interface systems (ALI), although also here dispersion in small droplets may sometimes be a preferred route of exposure. Despite the obvious benefits of exposing the biological system using dry aerosol, this also comes with drawbacks, such as the need for high amounts of test material, dedicated dosimetry studies, and/or adaptation of the exposure conditions (*e.g.*, duration, length, humidity and air flow). To facilitate reliable data interpretation, it is important to ensure stability under the exposure conditions. However, other methods may benefit from the fact that micron-sized particles eventually settle, *e.g.*, onto bronchial cell culture models, or are ingested by macrophages after deposition. In any case, the dispersion needs to be designed in such a way that it best mimics the expected exposure situation, to facilitate translation to "real-life" scenarios (including *in-vitro* to *in-vivo* extrapolations).

For environmental aqueous media, guidance is available for difficult test chemicals (OECD 2019), but this does not sufficiently cover difficulties with particles or particles in products. Therefore, for nanomaterials, OECD TG 318 has been developed, which provides methodology to disperse nanomaterials in aqueous media as well as methodology to determine the stability of such dispersions (OECD 2017). For media relevant to human health testing, similar methodologies may work, but these methodologies need to be scrutinised further to understand the impacts on the test material and the behaviour of AdMa in different environments (*i.e.*, river water vs. body fluids).The specific behaviour of multi-component AdMas may show further challenges in sample preparation (see Recommendation 3), which may also require adaptation of (some of) the methods developed or adapted for nanomaterials.

Within the MACRAMÉ project, SOPs have been developed for filtering respirable particles from aerosols to allow further testing. This also includes methods for sampling relevant subfractions, the different steps of detaching particles from filters, and (potential) necessary fractionation for further testing. The work also includes the comparison of different exposure devices for Air-Liquid-Interface methods, aiming at understanding the boundaries and advantages of each method. These steps may also influence the material itself, *i.e.*, the particles in the air may differ from particle samples detached from filters and/or fractionated and must be carefully considered in the light of the applicability of the intended testing system (*e.g.*, lung model for assessment of inhalation exposure).

Output / product(s) envisioned and action(s) required

The guidance on sample preparation for safety testing of AdMas should specify the required steps for the respective categories of AdMa, specific test, biological system, and/or endpoint.

In order to develop such a guidance, SOPs need to be developed for the adequate sample preparation in the media needed for the different safety testing methods required for AdMas. The guidance should link to existing OECD documents (including the recently published Guidance on Sample Preparation and Dosimetry for the Safety Testing of Manufactured Nanomaterials, (OECD 2025) and ISO standards and highlight the special needs for the appropriate sample preparation of AdMas, in addition or contrast to those for nanomaterials.

References

OECD (2012): Guidance on Sample Preparation and Dosimetry for the Safety Testing of Manufactured Nanomaterials. Edited by OECD Series on the Safety of Manufactured Nanomaterials and other Advanced Materials. OECD Publishing. Paris. Available online at https://doi.org/10.1787/ed430e1d-en.

OECD (2017): Test No. 318: Dispersion Stability of Nanomaterials in Simulated Environmental Media. Edited by OECD Guidelines for the Testing of Chemicals, Section 3. OECD Publishing. Available online at https://doi.org/10.1787/9789264284142-en.

OECD (2019): Guidance Document 23 on Aquatic Toxicity Testing of Difficult Substances and Mixtures. Edited by OECD Series on Testing and Assessment. OECD Publishing. Available online at https://doi.org/10.1787/0ed2f88e-en.

OECD (2025): Guidance on Sample Preparation and Dosimetry for Manufactured Nanomaterials. OECD Series on the Safety of Manufactured Nanomaterials and other Advanced Materials. Edited by Paris OECD Publishing. Available online at https://doi.org/10.1787/87ec4ecc-en.

6 Controlling and Describing the Dosimetry of Advanced Materials

Recommendation

Develop guidance on controlling and measurement of the dosimetry for adequate and accurate *in vitro* and *in vivo* testing for human and environmental toxicity of AdMas with realistic and relevant particle doses.

Issue

Meaningful *in vitro* experiments with lung cell models to assess the hazard potential of AdMas require the sound description of relevant doses. Administered dose, delivered dose and, if possible, also cellular doses (potentially in different cell types) need to be described using state-of-the art analytical methods (see (Schmid and Cassee 2017) for the definition of these dose metrics).

State-of-the-art and resources to be considered

Unlike well-defined particles, many AdMas exhibit complex morphologies and/or large (and sometimes multi-modal) size distributions. They are also subject to physical and chemical alterations during their life span. Researchers need to carefully consider this complexity before selecting the most relevant *in vitro* experiments mirroring possible routes of exposure. The exposure situation, and the possible uptake of AdMa particles into cells should be qualitatively described using appropriate microscopic imaging methods suitable to detect the particles in question. Also, for lung cell models, which were a major topic in MACRAMÉ, the influence of barrier structures such as mucus or lung surfactant as found in some evolved bronchial or lung models should be taken into account. Additionally, 3-dimensional cultures exist that mimic target tissue structures such as the lung parenchyma and these may require further evolved quantitative methods to describe particle uptake by different cell types (*e.g.*, epithelial plus phagocytic cells). Wherever possible, quantitative uptake data should be made available.

Dosimetry can affect the interpretation of hazard effects. In any test system, the administered dose of an AdMa should range from the no-effect level up to a reasonable worst-case scenario, whereby the decisive factor is the delivered dose that actually reaches the cells or test organism, and this should be explicitly determined or modelled.

In case of inhalation exposure, aerosol application is also an option and different model systems for the upper (bronchial) and lower airways (alveolar) may be used to investigate the toxicity of inhalable or respirable particles, respectively. When AdMas are to be tested for inhalation toxicity *in vitro*, inhalable or respirable particulates should be made available to competent cell cultures, *e.g.*, as particle suspensions or as dry powder aerosols. The most common and regulatory-relevant dose metric is still mass over surface, although, depending on the mode of action, effects of materials may primarily be driven by particle number and available surface area. In those cases, dose may be more appropriately expressed as a function of particle size, particle number, and/or specific surface area or volume. A reasonable dosing of AdMas to assess a potential hazard may be derived from the lung burden typically found in inhalation studies (*e.g.*, according to OECD TG 413 (OECD 2018)). It is very difficult to 1) assess the local dose in the lungs, and 2) relate that to specific cell types. As most *in vitro* systems mimic only a fraction of the lung surface/cell composition/morphology, there should be emphasis on how to go about translating exposure in intact lungs, to the specific area that is tested in an *in vitro* test

system. Dosimetry is also an issue in the case of ecotoxicity experiments, especially when prolonged exposure periods are used. Similar to human cell culture testing, it is necessary to consider that AdMas stability may be challenging in ecotoxicity exposure media (such as freshwater, marine water, or cell culture medium). Hence, accurately assessing the delivered dose (*i.e.*, the actual exposure dose received by cells or organisms) is essential to avoid both overestimation and underestimation of assay outcomes. Therefore, it is recommended to quantify the delivered dose throughout the exposure period (*e.g.*, at 24, 48, or 72 hours). In MACRAMÉ, methods for detecting graphene in cells were developed (MACRAMÉ Deliverable D2.8).

Output / product(s) envisioned and action(s) required

A better guidance on a dosimetry oriented towards a realistic particle dose and distribution of AdMas in human and animal tissues is needed as this will increase the predictability and acceptance of upcoming *in vitro* new approach methodologies (NAMs). The quantitative description of delivered and cellular dose is one major key aspect especially for single and repeated dose applications, which may become possible with suitable cell culture models. The guidance should clarify when to use surface area, particle number or other metrics.

The further development of quantitative high-resolution methods based on a combination of microscopic, analytical, and (mass) spectroscopic methods is needed to cover the growing complexity of AdMa analyses (see Recommendation 2).

References

OECD (2018): Test No. 413: Subchronic Inhalation Toxicity: 90-day Study. Edited by OECD Guidelines for the Testing of Chemicals, Section 4. OECD Publishing. Paris. Available online at https://doi.org/10.1787/9789264070806-en.

Schmid, Otmar; Cassee, Flemming R. (2017): On the pivotal role of dose for particle toxicology and risk assessment: exposure is a poor surrogate for delivered dose. In *Particle and fibre toxicology* 14 (1), p. 52. DOI: 10.1186/s12989-017-0233-1.

7 Testing and Assessment Strategies for Profound Risk Assessment of Advanced Materials Depending on Identified Exposure Points

Recommendation

Develop an Integrated Approach to Testing and Assessment (IATA) for AdMas that guides risk assessors towards essential tests to be performed on an AdMa and/or AdMa-containing product, depending on and considering the relevant identified exposure points as well as considering the AdMa's and/or product's associated fate and mobility.

Issue

The large number of different AdMas being produced represents a real challenge to ensure all are safe and fulfil regulatory obligations. Furthermore, assessment of their safety and sustainability requires that the life cycle of AdMas and/or AdMa-containing products are taken into account. This requires a more effective approach based on probabilities of release to prioritise testing as it will be impossible to test each and every material in each step in their life cycle.

State-of-the-art and resources to be considered

For nanomaterials the issue was already identified that it will be impossible to test each and every nanoform for hazard identification or safety assessment. To help tackle this issue, the GRACIOUS project developed different IATAs representing different exposure routes to guide and implement testing in a meaningful and cost-effective way. In addition to defining relevant exposure routes, an effective assessment strategy must explicitly include hazard identification as a core component. For AdMas, hazard identification poses major challenges compared to conventional chemicals and nanomaterials, due to their potential for transformation, complex composition, and emergent properties. These features may lead to novel mechanisms of toxicity that are not captured by standard test guidelines. Therefore, identifying relevant hazard endpoints early in the IATA framework is essential to ensure that subsequent testing adequately reflects the unique behaviour and risk profile of AdMas. An IATA will provide a stepwise approach to testing, which is cost effective while eventually addressing regulatory endpoints. It allows initial grouping of nanoforms based on physico-chemical properties (*e.g.*, dissolution), which will inform a stepwise testing strategy towards the more complex (regulatory) endpoints (Stone et al. 2020). It can help focus the assessment, *e.g.*, by first using test systems that mimic the various human biological barriers to allow uptake predictions and identify most relevant exposure routes.

For environmental endpoints, fate testing can help identify which environmental compartment is of greatest concern. Life cycle assessments can further inform the IATAs by identifying relevant exposure points in the life cycle, as such identifying the starting point of an IATA. Based on a generalised approach (Murphy et al. 2023), the GRACIOUS project developed 17 IATAs to address human hazard and 23 IATAs to address environmental hazards (Cross et al. 2024). These can serve as starting points for the development of IATAs for AdMas. The IATAs represent different exposure scenarios covering inhalation, ingestion, dermal, aqueous, soil, sediment and air. Information needs are generally overlapping and followed a similar approach. The HARMLESS project has developed tools to incorporate such approaches into safe-and-sustainable-by-design (Dekkers et al. 2025). Test method developments in MACRAMÉ can inform decisions in IATAs and contribute to their further development for next generation risk assessment of AdMas.

Output / product(s) envisioned and action(s) required

The envisioned outcome is one or a series of endpoints specific IATAs for AdMas that guides risk assessors effectively towards essential tests needed to characterise and evaluate AdMas and/or AdMa-containing products. The IATA(s) will take into account the relevant identified exposure points and subsequent fate and mobility of the (transformed) AdMa(s) assessed.

Additionally, a set of reliable and robust standards and reference materials for toxicity testing should be developed and made available, as has previously been done for nanomaterials via the JRC's repository of representative nanomaterials.

References

Cross, Richard K.; Spurgeon, Dave; Svendsen, Claus; Lahive, Elma; Little, Simon; Kammer, Frank et al. (2024): An integrated approach to testing and assessment (IATA) to support grouping and read-across of nanomaterials in aquatic systems. In *Nano Today* 54, p. 102065. DOI: 10.1016/j.nantod.2023.102065.

Dekkers, Susan; Adam, Veronique; Battista, Veronica de; Haase, Andrea; Prinz, Julia; Nagel, Gregor et al. (2025): Safe-and-Sustainable-by-Design Approach and Decision Support System for Advanced Materials. In *Authorea Preprints*. DOI: 10.22541/au.174077018.85147151/v1.

Murphy, Fiona A.; Johnston, Helinor J.; Dekkers, Susan; Bleeker, Eric A. J.; Oomen, Agnes G.; Fernandes, Teresa F. et al. (2023): How to formulate hypotheses and IATAs to support grouping and read-across of nanoforms. In *ALTEX* 40 (1), pp. 125–140. DOI: 10.14573/altex.2203241.

Stone, Vicki; Gottardo, Stefania; Bleeker, Eric A.J.; Braakhuis, Hedwig; Dekkers, Susan; Fernandes, Teresa et al. (2020): A framework for grouping and read-across of nanomaterials- supporting innovation and risk assessment. In *Nano Today* 35, p. 100941. DOI: 10.1016/j.nantod.2020.100941.

8 Identify Critical Steps in the Life Cycle where (Transformed) Advanced Materials are Released

Recommendation

Develop guidance on an optimised approach to identify where (transformed) AdMas are released along their life cycle to inform a more dedicated risk assessment.

Issue

For sustainability assessment, a closer look at the entire life cycle of products containing AdMas is essential. Such an assessment may already inform design issues related to the first steps in manufacturing an AdMa. Furthermore, identifying critical steps in the life cycle where (transformed) AdMas are released from products will inform and shape the assessment for products containing AdMas as part of the Safe and Sustainable by Design (SSbD) Framework. Guidance is needed on a material flow analysis approach that can effectively identify such critical steps.

State-of-the-art and resources to be considered

To determine whether a new product is a safer and more sustainable alternative compared to an existing AdMa or an AdMa-containing product, an analysis of its entire life cycle is required. An improvement in one area, for example, reduced use of mineral resources during manufacturing, might be overshadowed by an increase in human toxicity at the product's end-of-life stage.

The energy and materials requirements along the entire life cycle of the product will depend on the different processes applied in each phase of the life cycle, the machinery used, and the chemical properties of the materials. Moreover, during the different phases of the life cycle, there will be losses of materials and/or chemicals into water, air and/or soil, either directly from the AdMa, from transformed forms of the AdMa, or from its product matrix. The impact of these losses depends on the properties of the materials, the substances that compose them, and the routes of exposure resulting from their release.

Note that predicted release concentrations may be below current limits of detection. For example, for fibrous nanomaterials such as carbon nanotubes, the proposed occupational exposure limit (OEL) is 0.01 fibres/cm^3). With current transmission electron microscopy capabilities, evaluating such concentrations would require the analysis of more than 10^5 micrograph images, which is not feasible. Therefore, a dedicated risk assessment for the specific material or chemical in the relevant environmental compartment may be necessary, potentially requiring parallel analytical advances and further targeted toxicity testing (see also Recommendations 1-3).

To understand and predict release hot-spots relevant for risk assessment, it is important to define the material flows that might take place during the life cycle phases of a new product and to incorporate these into a testing concept (Adam et al. 2018). A material flow analysis (MFA) can be performed to represent these flows, making use of dynamic probabilistic models (Hauser and Nowack 2021; Hong et al. 2022). Such an analysis will help to identify the different AdMa forms and materials released as well as the environmental compartments into which they are emitted (Gilbertson et al. 2015; Rajkovic et al. 2020). Their environmental fate provides further insights into where and how to obtain the data

needed for the risk assessment. This, in turn, helps to focus the risk assessment on the most critical steps in the AdMas' and AdMas-containing products' life cycle.

A critical first step in conducting an MFA is to clearly define the system boundary and scope of the study. This will then inform the exact approach to be used. Approaches may include, for example, a static probabilistic model (not considering stocks) or a dynamic probabilistic model (considering the dynamics of the system and material stocks in specific compartments along the life cycle). Clear guidance on the performance and interpretation of MFA is needed to ensure consistency and to enable its broader application in risk assessment and within the SSbD framework. To update and validate model estimations on form-specific release (i.e. in what form AdMas are released from products at specific stages of their life cycle), experimental release testing should also be performed. For such testing, existing ISO standards for nanomaterials may form a starting point (*e.g.* (Wohlleben et al. 2024; ISO/TR 22293:2021; ISO/TS 12025:2021).

Output / product(s) envisioned and action(s) required

Guidance is envisioned on the best approach to identify transformation and release steps for (products containing) AdMas along their life cycle, to enable a systematic assessment of where emissions occur and in what form the AdMa is released, reflecting which transformation have taken place at the point of emission. This should include guidance on conducting MFA studies as a pre-experimental approach to pinpoint form-specific releases of the material under investigation from the specific product application, including criteria for selecting the approach (static versus dynamic) and for reporting and interpreting the outcomes. Such guidance would help derive simplified released masses or concentrations of the specific released form, which would in turn guide the user towards the environmentally relevant (eco)toxicity experiments to be applied in the environmental risk assessment.

References

Adam, Véronique; Caballero-Guzman, Alejandro; Nowack, Bernd (2018): Considering the forms of released engineered nanomaterials in probabilistic material flow analysis. In *Environmental pollution (Barking, Essex : 1987)* 243 (Pt A), pp. 17–27. DOI: 10.1016/j.envpol.2018.07.108.

ISO/TR 22293:2021, 2021: Evaluation of methods for assessing the release of nanomaterials from commercial, nanomaterial-containing polymer composites. Available online at https://www.iso.org/standard/73049.html#lifecycle.

Gilbertson, Leanne M.; Wender, Ben A.; Zimmerman, Julie B.; Eckelman, Matthew J. (2015): Coordinating modeling and experimental research of engineered nanomaterials to improve life cycle assessment studies. In *Environmental Science: Nano* 2 (6), pp. 669–682. DOI: 10.1039/C5EN00097A.

Hauser, Marina; Nowack, Bernd (2021): Probabilistic modelling of nanobiomaterial release from medical applications into the environment. In *Environment International* 146, p. 106184. DOI: 10.1016/j.envint.2020.106184.

Hong, Hyunjoo; Part, Florian; Nowack, Bernd (2022): Prospective Dynamic and Probabilistic Material Flow Analysis of Graphene-Based Materials in Europe from 2004 to 2030. In *Environmental science & technology* 56 (19), pp. 13798–13809. DOI: 10.1021/acs.est.2c04002.

ISO/TS 12025:2021, 2021: Nanomaterials — Quantification of nano-object release from powders by generation of aerosols. Available online at https://www.iso.org/standard/73131.html.

Rajkovic, Sana; Bornhöft, Nikolaus A.; van der Weijden, Renata; Nowack, Bernd; Adam, Véronique (2020): Dynamic probabilistic material flow analysis of engineered nanomaterials in European waste treatment systems. In *Waste management (New York, N.Y.)* 113, pp. 118–131. DOI: 10.1016/j.wasman.2020.05.032.

Wohlleben, Wendel; Bossa, Nathan; Mitrano, Denise M.; Scott, Keana (2024): Everything falls apart: How solids degrade and release nanomaterials, composite fragments, and microplastics. In *NanoImpact* 34, p. 100510. DOI: 10.1016/j.impact.2024.100510.

9 Standardised Release Testing of Advanced Materials along their Life Cycle

Recommendation

Develop standardised release tests that adequately mimic relevant exposure scenarios along AdMa life cycles to enable qualitative and quantitative analysis of (potential) releases from AdMas and AdMas-containing products along their life cycle. This should include detection and characterisation methods for released (fragments of) materials.

Issue

Standardised release tests adequately mimicking relevant exposure scenarios are needed for a comparable qualitative and quantitative analysis of fragments or droplets (potentially) released from AdMa-containing products along their life cycle. Thorough analysis of the released fragments in terms of their composition and physico-chemical characteristics that is suitable to the material properties of the specific AdMas and AdMa-containing products, should be performed subsequently to the release test. At the moment, not enough standards are available to cover the testing of AdMas along their life cycle.

State-of-the-art and resources to be considered

Exposure models are important tools for (regulatory) exposure assessment for diverse populations (*e.g.*, workers, consumers, elderly, etc.). Many of these models require release test data as input (Kuhlbusch et al. 2018; OECD 2015). Release of AdMas, or one or more of their components, along their life cycle is becoming increasingly important for sustainability assessment and for safety-by-design approaches. Understanding of the released forms is essential to correlate with any adverse outcomes, as the pristine AdMa properties may not be sufficiently predictive.

Within exposure scenarios, several activities could lead to a release of (fragments or droplets containing) nanomaterials or other AdMas. These released materials need to be adequately analysed in order to identify whether these contain the respective AdMa or its constituents, a transformed version of the AdMa, the matrix material, transformed matrix material or mixtures of those (Koivisto et al. 2017). For exposure assessment as well as safety testing, detailed data about the number of released fragments and about their composition and morphology is required (Nowack et al. 2013; Nowack et al. 2016).

So far, the implementation of releases of AdMas is a challenge, especially release information that is specific for certain morphologies or components. The determination of the characteristics of released fragments requires adequate detection and analytical methods also considering the complex nature of fragments being composed of matrix material, AdMas or transformed versions of both. In the early AdMa / product design phases with low amounts of material available and not yet scaled-up production processes in place, release tests could provide the required information on release and potential exposure.

Only a few of the available standardised release tests are validated for nanomaterials, and none are validated or standardised for AdMas yet. For some activity processes no release test is established at all. An OECD WPMN Guidance informing about available release tests for nanomaterials is currently being developed.

Besides the release testing per se, methods for the collection, detection, quantification and characterisation of the released fragments/droplets need to be tailored to adequately assess AdMas and their transformed versions (see also Recommendation 8). Especially for multi-component materials and for carbon-based AdMas the detection and characterisation methods are yet to be evaluated for their applicability.

In MACRAMÉ some of the processes in the life cycle methodologies were explored for use in release testing of AdMas. The Taber Abrader was used for testing the release of GR2M from epoxy matrices. Cryo-milling was used to simulate recycling processes at the end-of-life of CNT-containing products and incineration was performed to mimic waste treatment at the end-of-life. Learnings from characterisation and (eco)toxicity testing will feed into the development and standardisation of new release tests.

Output / product(s) envisioned and action(s) required

The required outputs are:

1) a testing strategy to cover all relevant exposure and transformation scenarios along the AdMa product life cycle that is generic enough to cover all AdMas, but includes additional bespoke recommendations for specific AdMa families, and

2) standardised release tests including standardised methods for detection and characterisation of the released fragments or droplets.

In order to achieve the envisioned output, the following actions are required:

- Identification of specific intentional and unintentional transformation processes during the life cycle of (products containing) AdMas for which no release test currently exists.
- Further validation and standardisation of existing release tests, developed for nanomaterials or chemicals, for their suitability for use with AdMas.

References

Koivisto, Antti Joonas; Jensen, Alexander Christian Østerskov; Kling, Kirsten Inga; Nørgaard, Asger; Brinch, Anna; Christensen, Frans; Jensen, Keld Alstrup (2017): Quantitative material releases from products and articles containing manufactured nanomaterials: Towards a release library. In *NanoImpact* 5, pp. 119–132. DOI: 10.1016/j.impact.2017.02.001.

Kuhlbusch, Thomas A.J.; Wijnhoven, Susan W.P.; Haase, Andrea (2018): Nanomaterial exposures for worker, consumer and the general public. In *NanoImpact* 10, pp. 11–25. DOI: 10.1016/j.impact.2017.11.003.

Nowack, Bernd; Boldrin, Alessio; Caballero, Alejandro; Hansen, Steffen Foss; Gottschalk, Fadri; Heggelund, Laura et al. (2016): Meeting the Needs for Released Nanomaterials Required for Further Testing-The SUN Approach. In *Environmental science & technology* 50 (6), pp. 2747–2753. DOI: 10.1021/acs.est.5b04472.

Nowack, Bernd; David, Raymond M.; Fissan, Heinz; Morris, Howard; Shatkin, Jo Anne; Stintz, Michael et al. (2013): Potential release scenarios for carbon nanotubes used in composites. In *Environment International* 59, pp. 1–11. DOI: 10.1016/j.envint.2013.04.003.

OECD (2015): Harmonized tiered approach to measure and assess the potential exposure to airborne emission of engineered nano-objects and their agglomerates and aggregates at workplaces. In *Series on the Safety of Manufactured Nanomaterials* (No. 55. ENV/JM/MONO(2015)19).

10 Influence of Physical and Chemical Transformations in the Environment on Risk Profiles of Advanced Materials

Recommendation

Develop guidance on how to test the transformations of the physical form of AdMas occurring in the environment.

Issue

To what extent transformation of AdMas and/or their constituent parts following release alters the exposure and hazard profile of the material is an important question in risk assessment. It is currently not always clear what the most relevant transformation processes are (and thus what the relevant form for (eco)toxicological assessment is), or how these environmental transformations are influenced by the prior transformations in the product and the released form (*i.e.*, a hierarchy of transformations). Additionally, standardised methodologies may not always be available to determine and/or quantify these processes.

State-of-the-art and resources to be considered

Test methods exist to determine abiotic and biotic transformations of chemicals (*e.g.*, OECD TG 301 (OECD 1992a), OECD TG 302B (OECD 1992b), OECD TG 307 (OECD 2002a), OECD TG 308 (OECD 2002b), OECD TG 309 (OECD 2004)). These mainly aim to capture (bio)transformation processes that transform organic chemicals into different chemicals, ultimately often resulting in carbon dioxide and water. For particulate advanced (nano)materials, different environmental transformations become important beyond the chemical transformations that are currently the focus of standardised test methods. Not only different chemicals may be formed, but there may also be physical changes, *e.g.*, change in particle size, creating structural defects or loss/change of coating. To further complicate the issue other transformation processes may play a role as well, and these may occur under natural conditions, *e.g.*, through weathering or exposure to UV radiation. As noted also in Recommendation 3, the impacts of these transformations may be different on the different components of multi-component AdMas. As the physical characteristics may also influence the environmental fate and toxicity of particulate materials, it is crucial to monitor such transformations in test systems to assess environmental fate and toxicity. To facilitate this, a hierarchy of AdMa transformations is needed that reflects changes that can occur at each stage of the life cycle, including their reversibility or irreversibility, and the consequences of these early transformations on which environmental transformations can occur upon release. Additionally, consideration should be given to the boundaries beyond which the AdMa is no longer of concern, *i.e.*, to what extent by-products or degradation products of the AdMas are included in the risk assessment – this can build on work from the Horizon 2020 NanoFASE project which developed initial thinking in this direction (Svendsen et al. 2020).

Physical transformations are dynamic processes influenced not only by the intrinsic physico-chemical properties of AdMas, such as size, composition, surface area, surface charge, and morphology, but also by external factors like temperature, pH, ionic strength, and the presence of inorganic and organic constituents in the surrounding medium. A crucial first step in identifying the most relevant physical (and chemical) transformations of AdMas is, therefore, to define the environmental compartment where these transformations may occur based on where in the life cycle the release to the environment

occurs (see Recommendation 9). Physical transformations can vary significantly between soil, freshwater, and marine environments based on the local chemistry and available biomolecules.

In addition to processes like dissolution or (hetero)agglomeration (addressed in OECD GD 318 (OECD 2020)) also sulphidation in anoxic conditions (in soil/sediments) may lead to changes in physical properties (*e.g.*, change of particle size, loss of coatings, or affinity for biomolecules) (Stetten et al. 2022; Svendsen et al. 2020). Furthermore, biotic transformations may lead to physical changes in advanced (nano)materials, *e.g.*, when a protein or eco-corona is formed (Wheeler et al. 2021; Gharibi et al. 2024) or when bacteria use only a specific component of a multi-component AdMa as a carbon source.

For abiotic transformations of nanomaterials, a guidance document is under development in the OECD (Test Guideline Program Project 3.16 (OECD)), but for biotic transformations further work is still needed for nanomaterials, and especially also to identify to what extent current (bio)degradation test methods are applicable for AdMas (Malta Initiative 2024).

The OECD “Physical-Chemical Decision Framework to Inform Decisions for Risk Assessment of Manufactured Nanomaterials” (OECD 2019) may provide a starting point, but to fully address the transformation issue, further detailed stepwise approaches (*e.g.*, IATAs) may need to be developed.

Output / product(s) envisioned and action(s) required

As a first step, a scoping review should be performed to identify the most important transformation processes that may occur in each of the different environmental compartments in which AdMas may end up, and that influence physical and chemical changes relevant for the risk profiles of these materials. The organisation of these transformations into a hierarchy, linked to the AdMa and product life cycle and to the Material Flow Analysis described in Recommendation 8, will enable generalisation across AdMas as far as possible and may include identification of material-specific considerations (*e.g.*, (OECD 2025) recommends minimising EDTA concentrations in aqueous media when working with metal nanomaterials in ecotoxicity tests), potential blind spots in current test guidelines, or recommendations to avoid/mitigate these.

Subsequently, guidance documents and/or harmonised/standardised test methods (ISO/OECD) should be developed to reproduce these release/transformation scenarios and evaluate the transformation of advanced (nano)materials under controlled environmental conditions. Additionally, there is a need to consider the scientific basis of grouping and read-across, which for nanomaterials/AdMas is currently performed on the basis of the pristine characteristics, but these may not apply to the released (transformed) form.

Test methods may include modelling tools to predict physical transformation of advanced (nano)materials in different environmental compartments under defined environmental conditions (*e.g.*, pH, salinity, temperature, natural organic matter content) (Dale et al. 2015; Meesters et al. 2019). IATAs and any related guidance should also take these aspects into account.

References

Dale, Amy L.; Casman, Elizabeth A.; Lowry, Gregory V.; Lead, Jamie R.; Viparelli, Enrica; Baalousha, Mohammed (2015): Modeling nanomaterial environmental fate in aquatic systems. In *Environmental science & technology* 49 (5), pp. 2587–2593. DOI: 10.1021/es505076w.

Gharibi, Hassan; Ashkarran, Ali Akbar; Jafari, Maryam; Voke, Elizabeth; Landry, Markita P.; Saei, Amir Ata; Mahmoudi, Morteza (2024): A uniform data processing pipeline enables harmonized nanoparticle protein corona analysis across proteomics core facilities. In *Nature communications* 15 (1), p. 342. DOI: 10.1038/s41467-023-44678-x.

Malta Initiative (2024): Malta Initiative Priority List https://malta-initiative.org/what/#MI-Priority-List. https://malta-initiative.org/what/#MI-Priority-List. Available online at https://malta-initiative.org/what/#MI-Priority-List.

Meesters, J. A. J.; Peijnenburg, W. J. G. M.; Hendriks, A. J.; van de Meent, D.; Quik, J. T. K. (2019): A model sensitivity analysis to determine the most important physicochemical properties driving environmental fate and exposure of engineered nanoparticles. In *Environ. Sci.: Nano* 6 (7), pp. 2049–2060. DOI: 10.1039/C9EN00117D.

OECD: Guidelines for the Testing of Chemicals. Available online at https://www.oecd.org/en/topics/sub-issues/testing-of-chemicals/test-guidelines.html.

OECD (1992a): Test No. 301: Ready Biodegradability. Edited by OECD Guidelines for the Testing of Chemicals, Section 3. OECD Publishing. Paris. Available online at https://doi.org/10.1787/9789264070349-en.

OECD (1992b): Test No. 302B: Inherent Biodegradability: Zahn-Wellens/ EVPA Test. Edited by OECD Guidelines for the Testing of Chemicals, Section 3. OECD Publishing. Paris. Available online at https://doi.org/10.1787/9789264070387-en.

OECD (2002a): Test No. 307: Aerobic and Anaerobic Transformation in Soil. Edited by OECD Guidelines for the Testing of Chemicals, Section 3. OECD Publishing. Paris. Available online at https://doi.org/10.1787/9789264070509-en.

OECD (2002b): Test No. 308: Aerobic and Anaerobic Transformation in Aquatic Sediment Systems. Edited by OECD Guidelines for the Testing of Chemicals, Section 3. OECD Publishing. Paris. Available online at https://doi.org/10.1787/9789264070523-en.

OECD (2004): Test No. 309: Aerobic Mineralisation in Surface Water – Simulation Biodegradation Test. Edited by OECD Guidelines for the Testing of Chemicals, Section 3. OECD Publishing. Paris. Available online at https://doi.org/10.1787/9789264070547-en.

OECD (2019): Physical-Chemical Decision Framework to inform Decisions for Risk Assessment of Manufactured Nanomaterials. OECD Series on the Safety of Manufactured Nanomaterials and other Advanced Materials. Edited by OECD Series on the Safety of Manufactured Nanomaterials and other Advanced Materials. OECD Publishing. Paris. Available online at https://doi.org/10.1787/1fbbaf8c-en.

OECD (2020): Guidance Document for the Testing of Dissolution and Dispersion Stability of Nanomaterials, and the Use of the Data for Further Environmental Testing and Assessment. OECD Series on Testing and Assessment, No. 318. Edited by OECD Publishing. Paris. Available online at https://doi.org/10.1787/f0539ec5-en.

OECD (2025): Guidance Document on Aquatic and Sediment Toxicological Testing of Nanomaterials, Second Edition, OECD Series on Testing and Assessment, No. 420,. Available online at https://doi.org/10.1787/17b3ce6f-en.

Stetten, Lucie; Hofmann, Thilo; Proux, Olivier; Landrot, Gautier; Kaegi, Ralf; Kammer, Frank von der (2022): Transformation of zinc oxide nanoparticles in freshwater sediments under oxic and anoxic conditions. In *Environmental Science: Nano* 9 (11), pp. 4255–4267. DOI: 10.1039/D2EN00709F.

Svendsen, Claus; Walker, Lee A.; Matzke, Marianne; Lahive, Elma; Harrison, Samuel; Crossley, Alison et al. (2020): Key principles and operational practices for improved nanotechnology environmental exposure assessment. In *Nature nanotechnology* 15 (9), pp. 731–742. DOI: 10.1038/s41565-020-0742-1.

Wheeler, Korin E.; Chetwynd, Andrew J.; Fahy, Kira M.; Hong, Brian S.; Tochihuitl, Jose A.; Foster, Lilah A.; Lynch, Iseult (2021): Environmental dimensions of the protein corona. In *Nature nanotechnology* 16 (6), pp. 617–629. DOI: 10.1038/s41565-021-00924-1.

11 Life Cycle Information as Part of Advanced Material Characterisation

Recommendation

Guidance for a provenance-based documentation of the AdMa life cycle, including its environment and covering information on each individual life cycle stage and transformations between stages, improving interpretability and comparability of safety assessments.

Issue

As AdMas may transform during their life cycle and use in products, a physico-chemical characterisation only at the beginning of its life cycle (pristine state) will often not be sufficient for its identification and for understanding possible exposure scenarios at later life cycle stages. However, a thorough characterisation of the material throughout its life cycle will often also not be feasible. Thus, required and practical chemical identification and physico-chemical characterisation (see Recommendations 1 and 2) should be enriched by a complementary description of the material and its state in the form of material, sample, and data provenance trails that document all handling steps of the AdMas and the data generated about them.

State-of-the-art and resources to be considered

Minimal (regulatory) information requirements and reporting standards provide a set of physico-chemical characteristics to uniquely identify a pristine AdMa (see Recommendation 1). However, for later life cycle stages, additional specific characterisation may be needed for certain material classes or products. Life cycle stages that considerably change the identity of the AdMas may include product formulation (intentional transformations) and use stage (unintentional transformations), end-of-life stage (degradation), and in some cases even the production stage.

To optimise hazard and risk assessment, an evaluation of sameness and similarity along the AdMa/product life cycle appears essential to allow meaningful grouping approaches and effective use of test results to correlate exposure with effect and to decide which life cycle stages need to be considered separately. *Sameness* refers to a material being indistinguishable from another material in terms of physico-chemical characterisation, producer, and specific batch. *Similarity* refers to materials that are sufficiently alike for read-across approaches, *i.e.*, using (hazard) data from one material to assess a compositionally similar material.

A thorough characterisation for each life cycle stage would dramatically increase the costs for identification and characterisation, as important aspects of creating the safety profile of an AdMa might not even be possible at all because of the many properties that may influence the safety profile or the lack of access to sufficient material quantities for testing.

Specifically for nanomaterials, OECD TGs and GDs have been or are currently being adapted or newly developed to enable their uniquely identification and characterisation. Additionally, minimal sets of characterisation parameters have been proposed (see Recommendation 10). These include a classification framework for graphene-based materials (see Recommendation 5) and requirements to characterise nanoforms for the REACH Regulation (EU Commission 2008).

Apart from testing just one species at a time in environmental safety testing, more complex multi-species mesocosm experiments can be performed that allow species interactions. To deal with this

complexity, first the NIKC database and later the EU projects NanoFASE and NanoCommons introduced a new way to characterise nanomaterials in their life cycle stages and in complex environments. Instead of relying solely on direct physico-chemical characterisation, materials are identified and characterised by reporting their history and fate, *i.e.*, all stages they have passed through during production and later modification (integration into product and/or biological matrices). This was visualised in so-called Instance Maps (Amos et al. 2024).

The MACRAMÉ project contributed to an updated version of this concept by describing Study Design Maps, which provide provenance information (who did what, when, and how to the material, and who generated what data about the material, when, and how) (Punz et al. 2025). For the MACRAMÉ case studies, these provenance trails cover the AdMa itself, its formulation into a product, and its preparation for toxicity or ecotoxicity assessment, as well as the resulting data generated using the AdMa or product, including all characterisation data. These Study Design Maps are supplemented by explicit descriptions of the transformation from one stage to the next (see Recommendation 12). These not only visually present the links between later and earlier life cycle stages but also allow the transformations to be documented in the (meta)data describing the material/test object.

Output / product(s) envisioned and action(s) required

A new data framework is needed that implements a dual approach for AdMa characterisation based on direct measurements of physico-chemical properties combined with a life cycle description of the AdMa. Such a life cycle description should include the history and fate of the material and its environment (*i.e.*, complex product and biological matrices) up to the life cycle stage under investigation. The description should also include information on the material's environment (*i.e.*, complex product and biological matrices). This should be captured in Findable, Accessible, Interoperable, and Reusable (FAIR) (meta)data as an extension of data provenance standards. Today's standards like PROV-O focus on digital transformations of data (*e.g.*, data curation, merging, analysis as data provenance trails) but work is on its way, *e.g.* started by WorldFAIR, to cover chemical and physical transformations of materials and samples (*e.g.*, raw material supply, production, transportation, use scenarios, sample preparation) to describe material and sample provenance.

Once established, the data framework can then be adopted as a standard (*e.g.*, ISO or CEN) or as an OECD Guidance providing:

- A minimal reporting checklist for physico-chemical characterisation of the current and previous AdMa-containing product life cycle stages, potentially combined with safety and sustainability aspects from earlier stages;
- options for metadata schemes covering material, sample, and data provenance trails describing life cycle stages and transformations between them.

Instance Maps and Study Design Maps could help to visualize and evaluate the complete life cycle and use scenarios and to associate potential risks with each of these scenarios. Guidance should also be developed on how to use this information for group-based risk assessments. Finally, the framework needs to be fully aligned with data management and sharing practices according to the FAIR principles. In addition, ways to store, connect, and access information from both earlier and later life cycle stages need to be defined (see Recommendation 12).

In this way, the data framework will constitute both a minimal reporting standard for chemical and material life cycle documentation beyond physico-chemical characterisation, as well as a harmonised and annotated (meta)data documentation standard for the unambiguous identification and characterisation of test objects at crucial life cycle stages.

This should take into account the information on previous life cycle stages and transformation steps between stages (*i.e.*, mapping to the transformation hierarchy and reconsidered basis for grouping of AdMas, see Recommendation 10), which would allow to extend risk assessment to the use and end-of-life stages.

References

Amos, Jaleesia D.; Zhang, Zhao; Tian, Yuan; Lowry, Gregory V.; Wiesner, Mark R.; Hendren, Christine Ogilvie (2024): Knowledge and Instance Mapping: architecture for premeditated interoperability of disparate data for materials. In *Scientific data* 11 (1), p. 173. DOI: 10.1038/s41597-024-03006-8.

EU Commission (2008): Regulation (EC) No 1272/2008. of the European Parliament and of the Council of 16 December 2008 on classification, labelling and packaging of substances and mixtures, amending and repealing Directives 67/548/EEC and 1999/45/EC, and amending Regulation (EC) No 1907/2006 (Text with EEA relevance). Available online at http://data.europa.eu/eli/reg/2008/1272/oj.

Punz, Benjamin; Brajnik, Maja; Dokler, Joh; Amos, Jaleesia D.; Johnson, Litty; Reilly, Katie et al. (2025): Instance maps as an organising concept for complex experimental workflows as demonstrated for (nano)material safety research. In *Beilstein journal of nanotechnology* 16, pp. 57–77. DOI: 10.3762/bjnano.16.7.

12 Sharing of Safety-relevant Information on Advanced Materials along the Life Cycle

Recommendation

Define improved digital management and sharing approaches with corresponding reporting and (meta)data standards for safety-relevant information on AdMas, including transformations along the life cycle, that ensure that the data are Findable, Accessible, Interoperable, and Reusable (FAIR).

Issue

Effective identification and characterisation of individual components and material in a multi-component material or a product and providing all required data on these components is challenging or even impossible (see Recommendation 11), particularly with the increasing demand to integrate sustainability considerations in safety (and sustainability) assessment. Therefore, the bulk of this characterisation should be performed not in the multi-component material or product stage but for the individual components and thus, responsibility for this characterisation has to be shared between the producer of the final product, the suppliers of the components and even the suppliers of the raw and starting materials. This requires effective sharing of information along the life cycle stages, which will also help to avoid unnecessary repetition of information collection, based on digital data sharing approaches and digital (meta)data standards.

State-of-the-art and resources to be considered

Information on early life cycle stages (*i.e.*, synthesis and production stages, including raw or pristine materials) becomes increasingly available. Sources of such information include chemical safety data sheets, chemical databases like PubChem and regulatory databases from *e.g.*, ECHA and US EPA. However, the data required for risk assessment are not easy to extract, since they are generally available in formats that are insufficiently machine actionable. This complicates their extraction out of a huge amount of irrelevant information and data. Additionally, the data may not be fully publicly available (only metadata or summary data is public with no clear workflow to ask for access to the data) and do not always provide the metadata to evaluate their regulatory relevance and reliability.

Digital product passports have been defined in EU legislation (EU Commission 2024) (Ecodesign for Sustainable Products Regulation, ESPR) and are currently under development for specific material markets. These are meant to provide information on the chemical composition and safe handling to stakeholders downstream in the value chain and concerned with later life cycle stages (*e.g.*, during production and recycling). However, it is currently not clear if and what safety and sustainability information will be required and whether the information will be provided in a harmonised way across all material sectors. Furthermore, questions remain about how data confidentiality will be managed to balance transparency with protection of proprietary information, for example through tiered access. There are also uncertainties regarding whether passports will be demanded for materials representing intermediate products in the value chain, and how this information from previous stages will be provided and integrated into the data for downstream stages.

Instance Maps and their derivative Study Design Maps have been developed to document the history and fate of materials in research environments (see Recommendation 11). In this way, they represent material, sample and data provenance trails. By linking information and data about specific "instances" of an AdMa (*i.e.*, at production, following incorporation into a product, following release during the

use or disposal phase, etc.) to the Study Design Maps, the full assessment of a specific life cycle stage can be constructed as the sum of the data generated for this stage in combination with the information on all previous life cycle stages and the corresponding AdMa transformations (Punz et al. 2025). Structuring data according to these maps has the advantage that information can already be prepared for early life cycle stages, *i.e.*, raw material provision, before transforming the materials into later stages, *i.e.* material production, has even started. This can then be used to define the additional characterisation needs for the later stages and realistic exposure scenarios at these stages. Additionally, the burden of data collection can be distributed between all players in a material value chain, which will become essential for a manageable risk assessment that integrates the product and end-of-life stages. To support the aggregation of all this information, the "MACRAMÉ knowledge management and sharing solutions" are a mixed system of centralised and distributed components linked together with Instance / Study Design Maps (MACRAMÉ D3.1: Information Hub).

Output / product(s) envisioned and action(s) required

Sharing tools should be further harmonised and standardised and guidance should be created on how to structure data collection and sharing along the AdMa value chain and life cycle. Such tools should include governance of responsibility sharing, while considering the needs of both research laboratories and commercial settings. These tools should be compliant with the FAIR principles and aligned with the objectives of the Common Data Platform for Chemicals. The guidance should address and assure interoperability between all this information. Minimal reporting and (meta)data standards as well as digital and secure data sharing options need to be defined to streamline the flow of characterisation and safety-related data along AdMa value chains. All information needs to be available in digital, easily accessible and machine-actionable formats and include sufficient details for unambiguous identification and characterisation, including any material transformation.

To ensure that the necessary data will be generated and reported, these data should be mandatory. While the ESPR defines Digital Product Passports, it fell short of defining a corresponding Digital Material Passport. Such a Digital Material Passport was proposed by projects such as the DigiPass project as the foundation for achieving circular economy goals by providing safety and sustainability data required for Digital Product Passports. The passports should be equipped with documentation and visualisation of the relationships between data points and life cycle stages, *e.g.*, in the form of Study Design Maps as easy-to-understand representation of the material flow and fate. This would allow the development of the required digital infrastructure and a corresponding governance model allowing to negotiate the sharing of confidential, commercial-relevant information and data.

These approaches should provide valuable background for later analyses and assessment of life cycle stages (product use and end-of-life). They should become the mechanism to report the data obtained with standards or OECD TGs.

References

EU Commission (2024): Regulation (EU) 2024/1781. of the European Parliament and of the Council of 13 June 2024 establishing a framework for the setting of ecodesign requirements for sustainable products, amending Directive (EU) 2020/1828 and Regulation (EU) 2023/1542 and repealing Directive 2009/125/EC (Text with EEA relevance). Available online at http://data.europa.eu/eli/reg/2024/1781/oj.

Punz, Benjamin; Brajnik, Maja; Dokler, Joh; Amos, Jaleesia D.; Johnson, Litty; Reilly, Katie et al. (2025): Instance maps as an organising concept for complex experimental workflows as demonstrated for (nano)material safety research. In *Beilstein journal of nanotechnology* 16, pp. 57–77. DOI: 10.3762/bjnano.16.7.
